# Ethanol preservation and pretreatments facilitate quality DNA extractions in recalcitrant plant species

**DOI:** 10.1002/aps3.11519

**Published:** 2023-06-01

**Authors:** Gabriel Johnson, Steven W. J. Canty, Isaac H. Lichter‐Marck, Warren Wagner, Jun Wen

**Affiliations:** ^1^ Department of Botany/MRC 166 National Museum of Natural History, Smithsonian Institution Washington D.C. 20560 USA; ^2^ Smithsonian Marine Station Fort Pierce Florida 34949 USA; ^3^ Working Land and Seascapes, Smithsonian Institution Washington D.C. 20013 USA; ^4^ Department of Ecology and Evolutionary Biology University of California Los Angeles, 612 Charles E. Young Dr. South Los Angeles California 90095 USA; ^5^ Department of Integrative Biology and Jepson herbarium University of California, Berkeley 1001 Valley Life Sciences Bldg. Berkeley California 94720 USA

**Keywords:** ethanol, herbarium, mangrove, *Rhizophora mangle*, tissue preservation, Vitaceae

## Abstract

**Premise:**

The preservation of plant tissues in ethanol is conventionally viewed as problematic. Here, we show that leaf preservation in ethanol combined with proteinase digestion can provide high‐quality DNA extracts. Additionally, as a pretreatment, ethanol can facilitate DNA extraction for recalcitrant samples.

**Methods:**

DNA was isolated from leaves preserved with 96% ethanol or from silica‐desiccated leaf samples and herbarium fragments that were pretreated with ethanol. DNA was extracted from herbarium tissues using a special ethanol pretreatment protocol, and these extracts were compared with those obtained using the standard cetyltrimethylammonium bromide (CTAB) method.

**Results:**

DNA extracted from tissue preserved in, or pretreated with, ethanol was less fragmented than DNA from tissues without pretreatment. Adding proteinase digestion to the lysis step increased the amount of DNA obtained from the ethanol‐pretreated tissues. The combination of the ethanol pretreatment with liquid nitrogen freezing and a sorbitol wash prior to cell lysis greatly improved the quality and yield of DNA from the herbarium tissue samples.

**Discussion:**

This study critically reevaluates the consequences of ethanol for plant tissue preservation and expands the utility of pretreatment methods for molecular and phylogenomic studies.

The isolation of high‐quality DNA, defined as DNA with minimal degradation and copurified contaminants, is increasingly important for next‐generation sequencing (NGS) methodologies, especially as single‐molecule long‐read technologies continue to advance (Amarasinghe et al., 2020). Over the past several decades of plant molecular research, DNA isolation using cetyltrimethylammonium bromide (CTAB)‐chloroform (Doyle and Doyle, 1987) or column‐binding kits have become universally accepted conventions (Chase and Hills, 1991); however, certain biomolecules and physiological conditions exhibited in the vast array of plant diversity have required modifications to be made to DNA‐extraction workflows. Most extraction protocol modifications are primarily focused on the cell lysis and downstream methods for differentially removing impurities from the DNA (e.g., Japelaghi et al., 2011; Kalendar et al., 2021).

Silica gel has long been used as the standard desiccation medium for preserving plant tissues for DNA extraction (Chase and Hills, 1991). This simple, inexpensive method quickly kills plant cells before they undergo apoptosis‐induced DNA hydrolysis and become filled with oxidative polyphenols as part of senescence and the wound response (Simeonova et al., 2000). Studies have shown that ethanol is an effective plant tissue desiccant for the in situ preservation of DNA for extraction (Murray and Pitas, 1996). While silica preservation is satisfactory for preserving the leaf tissue of most plant species, ethanol preservation and/or pretreatments are advantageous because ethanol inhibits hydrolytic enzymes and renders cell walls more readily homogenized. In the present study, plant leaf tissues are pretreated with ethanol to improve the quantity and quality of the extracted DNA.

Plant cells may contain a diversity of nuclease enzymes, some of which are not dependent upon divalent cation cofactors for their activation (Flournoy et al., 1996; Adams et al., 1999). These enzymes remain preserved in silica gel–dehydrated tissues and immediately hydrolyze nucleic acids upon rehydration, even in lysate solutions that contain EDTA and other divalent cation chelators (Murray and Pitas, 1996). For tissue specimens that require lysis incubations longer than 30 min, such endogenous nucleases will quickly degrade DNA quality. Fortunately, these cofactor‐independent nucleases become irreversibly denatured when dehydrated with ethanol (Adams et al., 1999; Akindele et al., 2011).

The plant cell wall is composed primarily of cellulose microfibrils which, when dehydrated, lose their innate elasticity, rendering the cells easily mechanically disrupted in a bead mill (Fang and Catchmark, 2014). Despite this, the cell wall is a complex matrix of numerous polysaccharides, proteoglycans, proteins, enzymes, and other macromolecules (Zhang et al., 2021), and some plants have leaf cell walls that do not become sufficiently friable after silica desiccation but become more brittle after an ethanol pretreatment (Akindele et al., 2011).

There is a common misunderstanding among plant systematists that exposing leaf tissue to ethanol destroys DNA. This myth probably arose from the use of ethanol to prevent fungal growth in plant specimens collected in tropical areas (Hodge, 1947; Smith, 1971). In this technique, the plant is only superficially covered with relatively low‐concentration ethanol (40–60% or even lower) to preserve the gross morphology, meaning that the internal tissues may still rot and leave the DNA degraded. This ethanol‐spraying technique is wholly different from preserving DNA directly in 96% ethanol or treating silica‐dried tissues with ethanol. In this study, the leaf tissue is preserved in ethanol to establish that ethanol does not degrade DNA in tissue collections and confirm previous reports that proteinase digestion is needed to isolate DNA from these ethanol‐preserved tissues (Flournoy et al., 1996; Murray and Pitas, 1996). As described in previous studies (Akindele et al., 2011), desiccated tissues were pretreated with ethanol to demonstrate how this technique improves DNA quality and yield.

## METHODS

**Figure 1:**
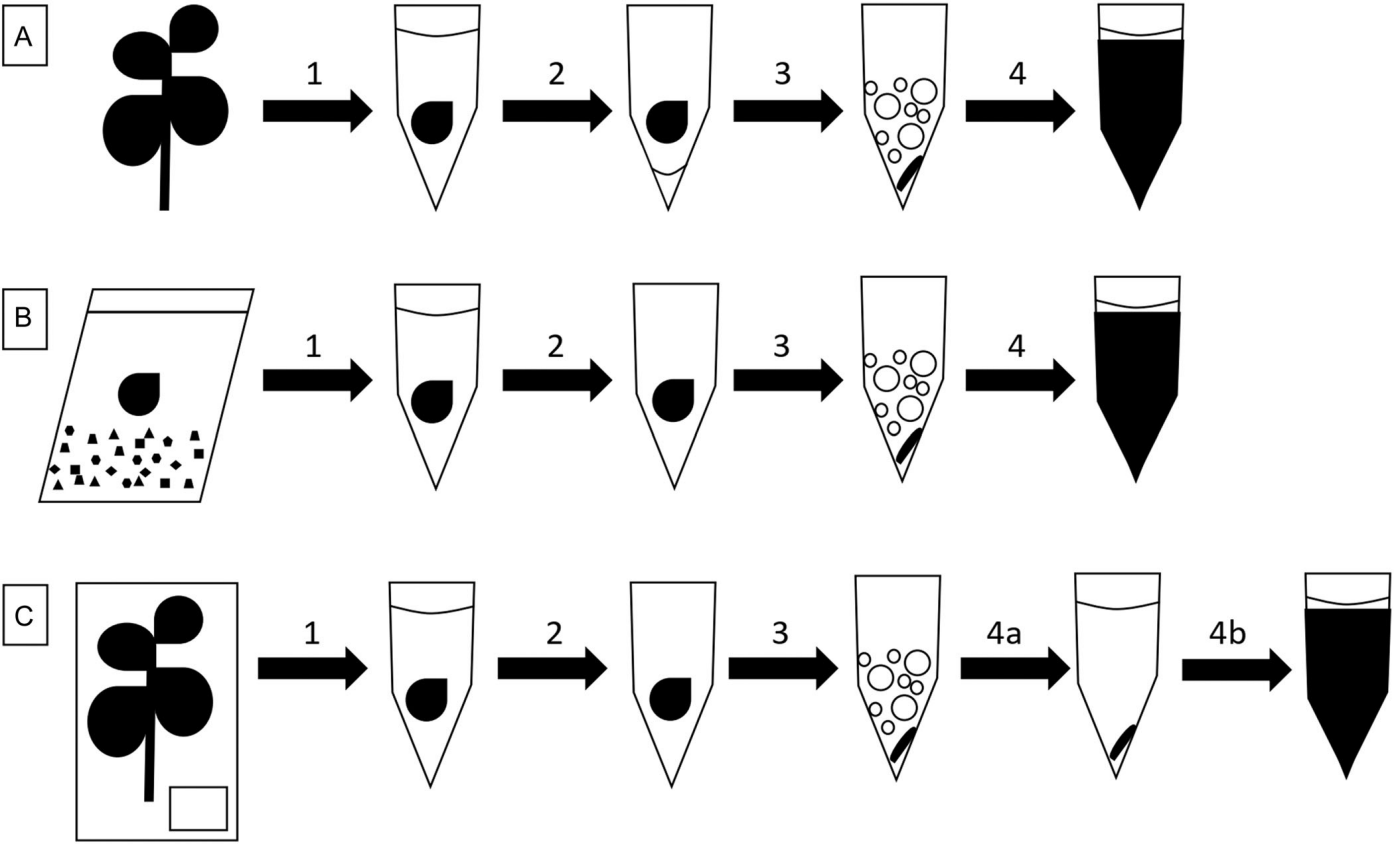
An overview of the three ethanol treatment experiments used in this study. (A) Leaf tissues from the Vitaceae species were immediately immersed in 96% ethanol after harvest and stored in this ethanol until they were homogenized in ethanol residue and used for DNA extraction. (B) *Rhizophora mangle* leaf tissues were desiccated in silica gel and then soaked in ethanol for several days. After the ethanol was removed and the residue evaporated, the tissue was homogenized and used for cell lysis and DNA extraction. (C) Herbarium specimen leaf fragments were soaked in ethanol for 2 h and homogenized after the ethanol was removed and the residue evaporated. The tissue homogenate was resuspended in sorbitol buffer and centrifuged to produce a tissue pellet that was subjected to cell lysis with proteinase K digestion. The same herbarium leaf samples were also extracted using the standard CTAB procedure (Doyle and Doyle, 1987). (1) Leaf tissue is placed into a tube of 96% ethanol and homogenization beads to incubate. (2) Ethanol is removed by pipetting and ethanol residue is left in tubes (A) or is allowed to evaporate (B and C). (3) Tissue is ground into a tissue powder without (A) or with freezing in liquid nitrogen (B and C). (4) Tissue homogenate is first washed with sorbitol buffer (4a in C) or is directly vortex‐mixed with SDS lysis buffer and additives (i.e., proteinase K and β‐mercaptoethanol). 4b shows vortexed lysate with SDS lysis buffer and additives.

Figure 1 presents an overview of the three experiments performed to test the effects of ethanol preservation and pretreatment on plant tissues. This figure shows the steps used before cell lysis and the DNA extraction procedure. The specific methodologies used in each experiment are described in the subsections below.

### Direct ethanol preservation

To test whether the ethanol‐based preservation of tissues would affect the quality of the DNA extracted, 13 species representing 10 Vitaceae genera (Table 1) were preserved in ethanol. The plants were grown in the Department of Botany research greenhouses at the National Museum of Natural History, Smithsonian Institution, Suitland, Maryland, USA. Only young expanding leaves were sampled, as is recommended for optimal DNA extraction (Mauro et al., 1992; Lodhi et al., 1994). The tissues were collected directly into 15‐mL Falcon tubes containing 12 mL of 96% ethanol. DNA was isolated from these ethanol‐preserved tissues at three weeks, six weeks, four months, and four years after harvesting. While incubating in ethanol, these samples were stored at room temperature in a dark flammables cabinet to protect the tissue from excessive light exposure.

**Table 1:** Taxon name, voucher number, and DNA concentration for the 13 samples collected and preserved in 96% ethanol. The DNA concentrations correspond to the DNA extracts prepared from each sample after three weeks, six weeks, and four years. Misplaced values are absent for the first two samples at the three‐week time point.

Sample taxon	Voucher	DNA concentration after 3 wk (ng/μL)	DNA concentration after 6 wk (ng/μL)	DNA concentration after 4 y^a^ (ng/μL)
*Causonis japonica* (Thunb.) Raf.	IB 2010‐082	NA	4.7	<0.01
*Tetrastigma lawsonii* Herb. Kew	Wen 11680	NA	5.0	<0.01
*Pterisanthes eriopoda* (Miq.) Planch.	Wen 11831	4.2	2.7	42.3
*Tetrastigma rafflesiae* Planch.	Wen 11824	37.2	28.3	710.0
*Ampelocissus ascendiflora* Latiff	Wen 11822	4.4	1.3	550.0
*Cissus discolor* Blume	IB 2012‐011	4.9	0.9	301.0
*Cyphostemma juttae* (Dinter & Gilg) Desc.	IB 2010‐093	4.8	2.8	407.0
*Cyphostemma sandersonii* (Harv.) Desc.	IB 2010‐094	6.7	2.8	183.0
*Cissus tuberosa* Moc. & Sessé ex DC.	IB 2010‐091	13.4	10.2	214.0
*Vitis monticola* Buckley	Wen 12662	2.1	3.4	137.0
*Vitis munsoniana* J. H. Simpson ex Planch.	Wen s.n.	4.1	11.0	174.0
*Parthenocissus heptaphylla* (Planch.) Britton	Wen 12657	4.2	7.7	71.8
*Nekemias arborea* (L.) J. Wen & Boggan	Wen 12694	9.0	16.0	291.0

^a^ After four years of storage, the DNA was extracted using proteinase K in the lysis buffer and a doubled amount of input tissue was processed.

For each of these four time points, a section of the leaf tissue approximating 1.0 cm^2^ was removed and rinsed with fresh 96% ethanol. While still covered with ethanol residue, the tissues were placed into 2.0‐mL screw‐cap tubes containing a mixture of ~50 µL of 1.0‐mm‐diameter glass and ten 2.5‐mm zirconium‐silica beads (BioSpec Products, Bartlesville, Oklahoma, USA). The tissues were homogenized in a MP FastPrep 96 (MP Biomedicals, Santa Ana, California, USA) for 1 min at 1800 rpm. Each homogenate was processed using a Qiagen DNeasy Plant Mini Kit (Qiagen, Hilden, Germany), according to the manufacturer's specifications, except for the four‐year samples for which 1.0 mg/mL total concentration of proteinase K and 2% (v/v) β‐mercaptoethanol were added to the lysis solution before the samples were incubated overnight at 54°C. At this last time point, twice as much input tissue was processed together.

### Ethanol pretreatment of silica‐desiccated samples

*Rhizophora mangle* L. (red mangrove) leaf tissue was collected from Fort Bay, within the Bay Island archipelago, Republic of Honduras, in April 2016 (Table 2) for use within a NGS population study (Canty et al., 2022). Due to the harsh collecting conditions and the large number of individuals sampled in this population genomic experiment, tissue harvesting directly into conical vials of ethanol was intractable. We collected two to three young leaves, which have lower concentrations of secondary metabolites (Kandil et al., 2004). These were collected from an individual mangrove, their petioles were removed, and the leaves were broken in half before being placed in individually labeled polyethylene resealable bags containing a 0.06–0.80‐mm granular mix of silica gel with a cobalt indicator. Although high‐quality *Bruguiera gymnorhiza* (L.) Lam. DNA was extracted by Huang et al. (2000), these authors used fresh leaf material which was unavailable here.

**Table 2:** DNA concentrations for *Rhizophora mangle* samples with and without ethanol pretreatment prior to homogenization and extraction.

No.	Sample	DNA concentration (ng/μL)	
		No ethanol pretreatment	Ethanol pretreatment
1	*Rhizophora mangle*	1.70	0.26
2	*Rhizophora mangle*	0.66	0.15
3	*Rhizophora mangle*	0.98	0.24
4	*Rhizophora mangle*	3.96	0.07
5	*Rhizophora mangle*	4.14	0.24
6	*Rhizophora mangle*	1.04	0.12
7	*Rhizophora mangle*	0.51	0.08
8	*Rhizophora mangle*	2.14	0.11
9	*Rhizophora mangle*	3.00	0.19
10	*Rhizophora mangle*	3.32	0.05
11	*Rhizophora mangle*	1.62	0.06
12	*Rhizophora mangle*	0.78	0.39
13	*Rhizophora mangle*	1.98	0.14
14	*Rhizophora mangle*	1.11	0.31
15	*Rhizophora mangle*	1.54	0.18
16	*Rhizophora mangle*	1.27	0.30
17	*Rhizophora mangle*	2.20	0.24
18	*Rhizophora mangle*	0.81	0.41
19	*Rhizophora mangle*	0.47	0.25
20	*Rhizophora mangle*	0.87	0.21

Leaf tissue fragments of about 1.0 cm^2^ were placed into screw‐cap tubes containing beads and were homogenized as described above. DNA was isolated from the tissue powder using the Qiagen DNeasy Plant Mini Kit, according to the manufacturer's protocol. Extractions without the ethanol pretreatment were conducted in December 2017. The same samples were then extracted again, except the tissue was first pretreated with ethanol in February 2018. After transferring the desiccated leaf fragments to the screw‐cap homogenization bead tube, 500 μL of 96% ethanol was added to completely immerse the tissue, followed by incubation for 4–5 days before the ethanol was removed. The lids of the tubes were left open for ~2 days at room temperature to allow the ethanol residue to fully evaporate, after which the tissues were ready to be lysed.

Regardless of the pretreatment, *R. mangle* leaf tissues were homogenized using a MM440 mixer mill (Retsch, Haan, Germany) with a single 5‐mm stainless steel ball bearing (Qiagen) at 30 Hz for 1 min, after which the samples were flipped and swapped between the mixer mill arms before being milled again at 30 Hz for 1 min. The DNA extraction was then conducted using a Qiagen DNeasy 96 Plant Kit, following the manufacturer's instructions.

### Ethanol pretreatment of herbarium tissue

Herbarium vouchers from several families with endemic Hawaiian taxa were destructively sampled from specimens in the United States National Herbarium (Table 3). The tissue fragments were divided in two, with DNA extracted from one half using the standard CTAB protocol (Doyle and Doyle, 1987) and from the other half using a modified procedure that incorporates an initial ethanol pretreatment. For this pretreatment, the tissue fragments were immersed in 96% ethanol for 2 h before most of the ethanol was removed by pipetting. The sample tubes were uncapped and covered with a Kimwipe (Kimberly‐Clark, Irving, Texas, USA) while incubated overnight in a fume hood to allow the ethanol residue to evaporate. The tissues were homogenized in a bead mill as described above. Cell lysis was conducted with CTAB for the samples that were not ethanol pretreated, or a sodium dodecyl sulfate (SDS) buffer for those that were. The SDS lysis buffer (0.5% SDS, 0.08 M NaCl, 0.16 M sucrose, 0.064 M EDTA, 0.12 M Tris base, pH 9.1) was used instead of CTAB because SDS stimulates the activity of proteinase K (Hilz et al., 1975) and subsequent precipitation with potassium acetate removes mucopolysaccharides (Sokolov, 2000). Each homogenized plant powder was resuspended in 500 µL of SDS buffer and vortex‐mixed with 10 µL of 50 mg/mL proteinase K and 10 µL of β‐mercaptoethanol before being incubated at 54°C overnight. Each sample received 5 µL of 20 mg/mL RNase A and was incubated at 65°C for 10 min before cooling to room temperature. Samples were then combined with 150 µL of 3 M potassium acetate (pH 4.7) and incubated on ice for 5 min. After centrifuging at 13,000 × *g* for 15 min, the supernatant was combined with 500 µL of 24:1 chloroform:isoamyl alcohol and mixed for 3 min. After centrifugation for 15 min, the upper aqueous phase was transferred to a new tube where it was mixed with a 0.08X volume of 7.5 M ammonium acetate and a 0.54X volume of isopropyl alcohol. The w/v concentration of ammonium acetate and isopropyl alcohol varied between samples depending on the volume of aqueous phase that was recovered. For example, if 500 µL of aqueous phase was recovered, then a 0.08X volume of added ammonium acetate (=40 µL) would be equivalent to a 0.0125% (w/v) total concentration. Subsequently, a 0.54X addition of isopropanol (=292 µL) to this 540 µL volume would make the final concentration of isopropanol 35.1% (v/v). The samples were incubated at −20°C overnight to precipitate the DNA, after which they were centrifuged for 20 min to pelletize the DNA. The pellets were washed twice with 1 mL of ethanol wash buffer (80% ethanol, 0.02 M NaCl, 0.002 M Tris‐HCl, pH 8.0), and after allowing the ethanol vapor to evaporate, the DNA was resuspended in 100 µL of TE buffer (0.01 M Tris‐HCl, 0.001 M EDTA, pH 8.0).

**Table 3:** Herbarium voucher specimens used in the comparison of DNA extraction efficacy using a standard CTAB procedure or a modified protocol that incorporates an ethanol pretreatment.

Family	Species	Variety	Voucher^a^	Collection date	Collection locality
Asteraceae	*Tetramolopium capillare* (Gaudich.) St. John		Perlman et al. 13760	16‐Sep‐93	USA, Hawaii, Maui
Asteraceae	*Tetramolopium rockii* Sherff		Wagner et al. 4910	29‐Jul‐83	USA, Hawaii, Molokai
Asteraceae	*Tetramolopium sylvae* Lowrey		Wood & LeGrande 9825	6‐Jun‐02	USA, Hawaii, Molokai
Cucurbitaceae	*Sicyos anunu* (H. St. John) I. Telford		Herbst 9779	3‐Jul‐96	USA, Hawaii, Hawaii
Cucurbitaceae	*Sicyos cucumerinus* A. Gray		Perlman et al. 22907	6‐Jun‐12	USA, Hawaii, Maui
Cucurbitaceae	*Sicyos hillebrandii* St. John		Oppenheimer H81402	12‐Aug‐14	USA, Hawaii, Maui
Euphorbiaceae	*Euphorbia celastroides* Boiss.	*amplectens* Sherff	Wood & Espainole 11116	26‐Jan‐05	USA, Hawaii, Molokai
Euphorbiaceae	*Euphorbia celastroides*	*laehiensis* O. Deg., I. Deg. & Sherff	Spence 308	18‐Oct‐73	USA, Hawaii, Lanai
Euphorbiaceae	*Euphorbia celastroides*	*celastroides*	Flynn & Harder 2996	9‐Jun‐88	USA, Hawaii, Kauai
Loganiaceae	*Geniostoma hedyosmifolium* (Baill.) Byng & Christenh.		Perlman et al. 23968	28‐May‐14	USA, Hawaii, Maui
Loganiaceae	*Geniostoma helleri* (Sherff) Byng & Christenh.		Perlman & Wood 15509	8‐Aug‐96	USA, Hawaii, Kauai
Loganiaceae	*Geniostoma triflorum* (Hillebr.) Byng & Christenh.		Lau 3421	18‐Aug‐91	USA, Hawaii, Molokai
Piperaceae	*Peperomia alternifolia* Yunck.		Wiebke & Nitta 3185	Aug‐28	USA, Hawaii, Molokai
Piperaceae	*Peperomia hypoleuca* Miq.		Wagner et al. 5975	13‐Mar‐88	USA, Hawaii, Hawaii
Piperaceae	*Peperomia mauiensis* Wawra		Wagner et al. 5835	6‐Mar‐88	USA, Hawaii, Maui
Pittosporaceae	*Pittosporum argentifolium* Sherff		Wood 10565	5‐Feb‐04	USA, Hawaii, Molokai
Pittosporaceae	*Pittosporum confertiflorum* A. Gray		Gemmill 328‐21	Mar‐96	USA, Hawaii, Hawaii
Pittosporaceae	*Pittosporum halophilum* Rock		Wood et al. 14464	26‐Jan‐11	USA, Hawaii, Molokai
Poaceae	*Panicum fauriei* Hitchc.	*carteri* (Hosaka) Davidse	Herbst 6104	24‐May‐78	USA, Hawaii, Oahu, Mokolii Islet
Poaceae	*Panicum fauriei*	*latius* (H. St. John) Davidse	Wood 11132	9‐Feb‐05	USA, Hawaii, Kauai
Poaceae	*Panicum pellitum* Trin.		Flynn et al. 5973	20‐Mar‐96	USA, Hawaii, Hawaii
Primulaceae	*Lysimachia forbesii* Rock		Degener 17689	22‐Jun‐32	USA, Hawaii, Oahu
Primulaceae	*Lysimachia maxima* (R. Knuth) H. St. John		Wood & Espainole 11361	23‐May‐05	USA, Hawaii, Molokai
Primulaceae	*Lysimachia remyi* Hillebr.		Wood 3212	20‐May‐94	USA, Hawaii, Maui
Rubiaceae	*Kadua axillaris* (Wawra) W. L. Wagner & Lorence		Wagner et al. 5870	8‐Mar‐88	USA, Hawaii, Maui
Rubiaceae	*Kadua formosa* Hillebr.		Perlman & Wood 16397	4‐Nov‐98	USA, Hawaii, Maui
Rubiaceae	*Kadua tryblium* (D. R. Herbst & W. L. Wagner) W. L. Wagner & Lorence		Wood 13458	29‐Jan‐09	USA, Hawaii, Kauai
Rubiaceae	*Psychotria greenwelliae* Fosberg		Wagner et al. 6045	9‐Apr‐88	USA, Hawaii, Kauai
Rubiaceae	*Psychotria greenwelliae*		Lorence et al. 10464	13‐Jul‐14	USA, Hawaii, Kauai
Rubiaceae	*Psychotria kaduana* (Cham & Schltdl.) Fosberg		Perlman 17479	18‐Jul‐17	USA, Hawaii, Kauai

^a^ Herbarium specimens are deposited at the United States National Herbarium, Washington, D.C., USA.

### Quality assessment

The degree of DNA fragmentation was determined using gel electrophoresis. For each sample, 2 μL of total DNA was mixed in a 1:1 ratio with a 1:1000 dilution of Gel Red (Biotium, Fremont, California, USA) in a glycerol‐based loading dye (Cold Spring Harbor Laboratory Press, 2007). The resulting 4‐μL solution was transferred into a 1.0% (w/v) agarose gel prepared with Seakem agarose LE (Lonza, Rockland, Maine, USA) and 1X sodium boric acid buffer (Brody and Kern, 2004). Electrophoresis was conducted at a constant 120 V for 2–4 cm separation. The size distribution of the bands was compared against the Fast DNA Ladder (New England Biolabs, Ipswich, Massachusetts, USA). The gel fluorescence was visualized and digital images were captured using the UVP Doc‐IT transilluminator system (Analytik Jena, Jena, Germany).

DNA concentrations were determined using a Qubit dsDNA broad range assay kit (Thermo Fisher Scientific, Waltham, Massachusetts, USA), according to the manufacturer's specifications. The exception to this was the quantification of DNA from the non‐ethanol‐pretreated *R. mangle* samples, for which a BioTek Epoch Microplate Spectrophotometer (Agilent Technologies, Santa Clara, California, USA) was used.

The statistical significance of associations between herbarium sample DNA yield or quality and the extraction approach were assessed using paired *t*‐tests, two‐tailed ANOVAs, and a linear regression analysis (Zar, 1996). All analyses were performed in the R statistical computing environment (R Core Team, 2014).

## RESULTS AND DISCUSSION

### Direct ethanol preservation does not prevent DNA extraction

**Figure 2:**
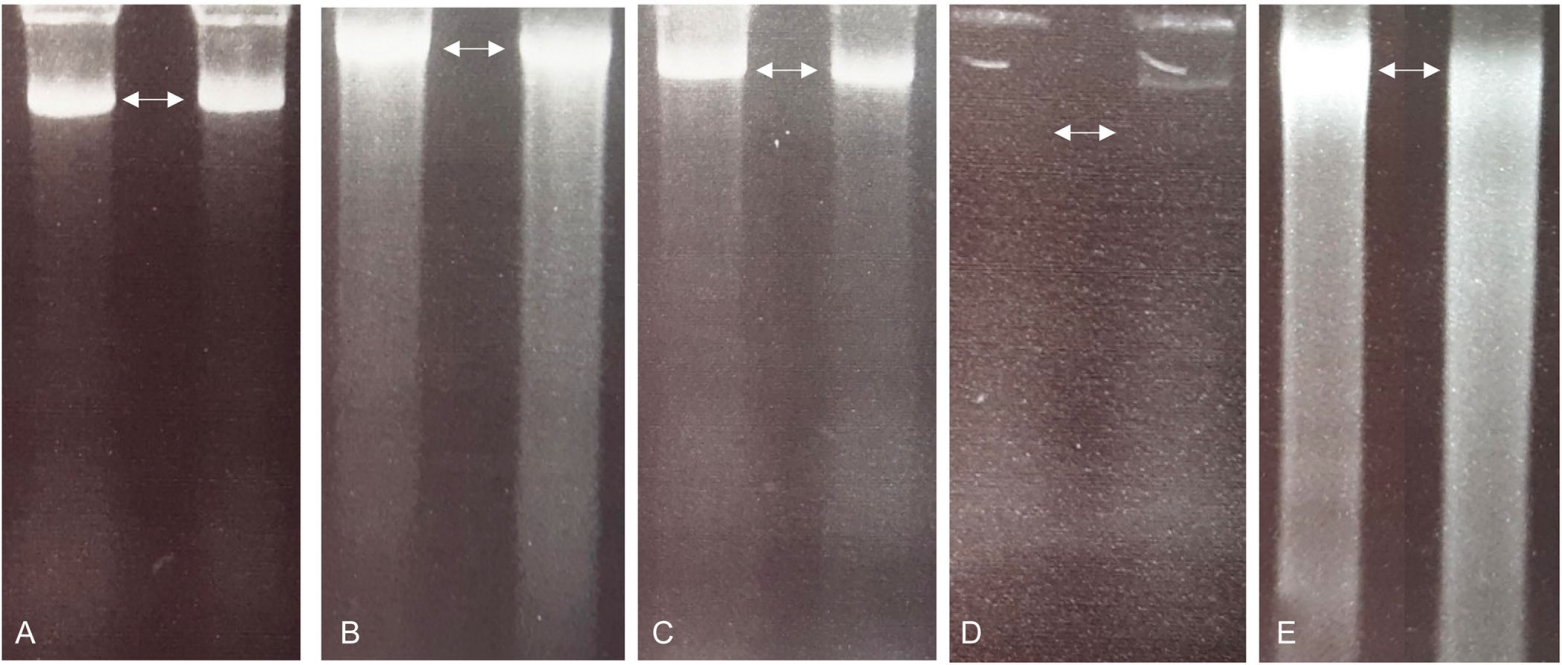
Agarose electrophoresis gel separations showing DNA extractions from ethanol‐preserved tissue for *Ampelocissus ascendiflora* on the left and *Cissus discolor* on the right of each image. The DNA extractions were performed after (A) three weeks, (B) six weeks, (C) four months, and (D) four years of storage in ethanol. An overnight proteinase K digestion was also used in the lysis of the four‐year group (E). DNA size ladder is not shown; however, each gel band indicated by arrows is parallel with the 10‐kbp maximum band. Gel separation data for the other taxa (not shown) had similar patterns.

Tissues wet with ethanol could be fully pulverized in a bead mill without requiring liquid nitrogen, and the resulting homogenate did not form a messy friable powder. After six weeks of incubation in ethanol, the DNAs isolated from the Vitaceae tissues had virtually the same quality as extracts prepared just after harvesting (Figure 2). After four months of ethanol storage, DNA was no longer obtainable for the majority of samples using the standard Qiagen DNeasy kit protocol (Table 1); however, the DNA was not destroyed. Rather, as suggested by Flournoy et al. (1996), the DNA was completely covered with dehydrated proteins that had been gradually congealing around the nucleic acids since the tissues were first submerged in ethanol preservative. After four years of preservation in ethanol, DNA could be obtained from almost all the samples following the addition of proteinase K to the lysis solution (Table 1). This may indicate that the dehydrated proteins were digested away from the nucleic acids, meaning DNA was not inadvertently discarded during the deproteination steps of the procedure.

### Ethanol pretreatment of silica‐desiccated samples results in higher‐quality DNA extractions

**Figure 3:**
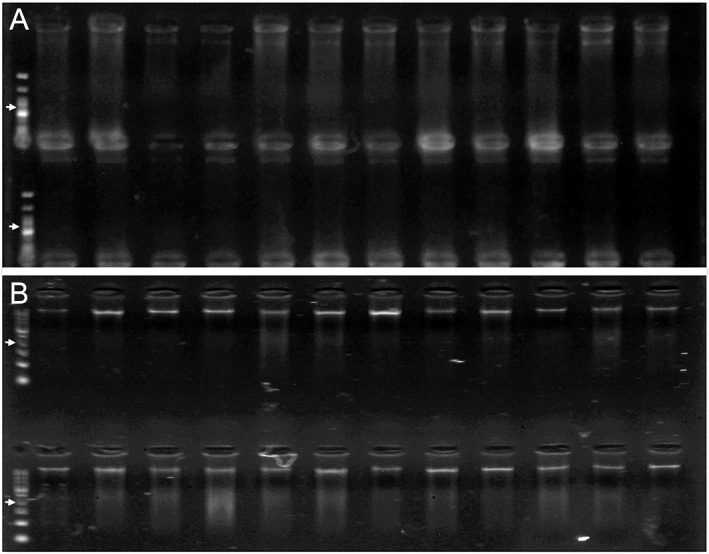
Gel electrophoresis images of the extracted DNA of 24 *Rhizophora mangle* (red mangrove) samples, either without (A) or with (B) an ethanol treatment. Different ladders were used for each extraction type. Arrows indicate the 300‐bp‐ladder band.

*Rhizophora mangle* tissue desiccated with silica gel yielded fragmented DNA; however, when this silica‐dried tissue was treated with ethanol a day before homogenization, the resulting DNA formed high‐molecular‐weight bands in the gel separation (Figure 3), albeit in much lower concentrations (Table 2). The mean DNA concentration with no ethanol treatment was 41.24 ± 3.12 ng/μL, compared with just 0.46 ± 0.03 ng/μL following the ethanol pretreatment, but the DNA extracted from the ethanol‐treated samples was of higher quality. The samples with no ethanol treatment failed during the 2b‐RAD library preparation following the modified protocol of Wang et al. (2012), whereas, after concentration, the ethanol‐treated samples could be used effectively to produce usable 2b‐RAD libraries (Canty et al., 2022). It is possible that a greater amount of DNA would have been obtained from the ethanol‐treated *R. mangle* leaves if a proteinase K digestion was included in the cell lysis step.

Mangrove leaves are naturally tough and resistant to desiccation (Feller, 1995). Here, however, the leaf tissue was noticeably more friable and easily homogenized into a fine powder after the ethanol treatment, as was reported for other species (Sharma et al., 2003; Linke et al., 2010). It was as though a key element of the structural polysaccharides had leached from the apoplast, causing an irreversible change in the wall elasticity, a change not observed in silica‐dried tissues. Ethanol has been proven to effectively extract secondary metabolites from mangrove leaves (Cruz et al., 2015), which may explain the altered structure of the pretreated samples.

### Ethanol pretreatment enhanced the yield and quality of DNA extracts from herbarium tissue

**Figure 4:**
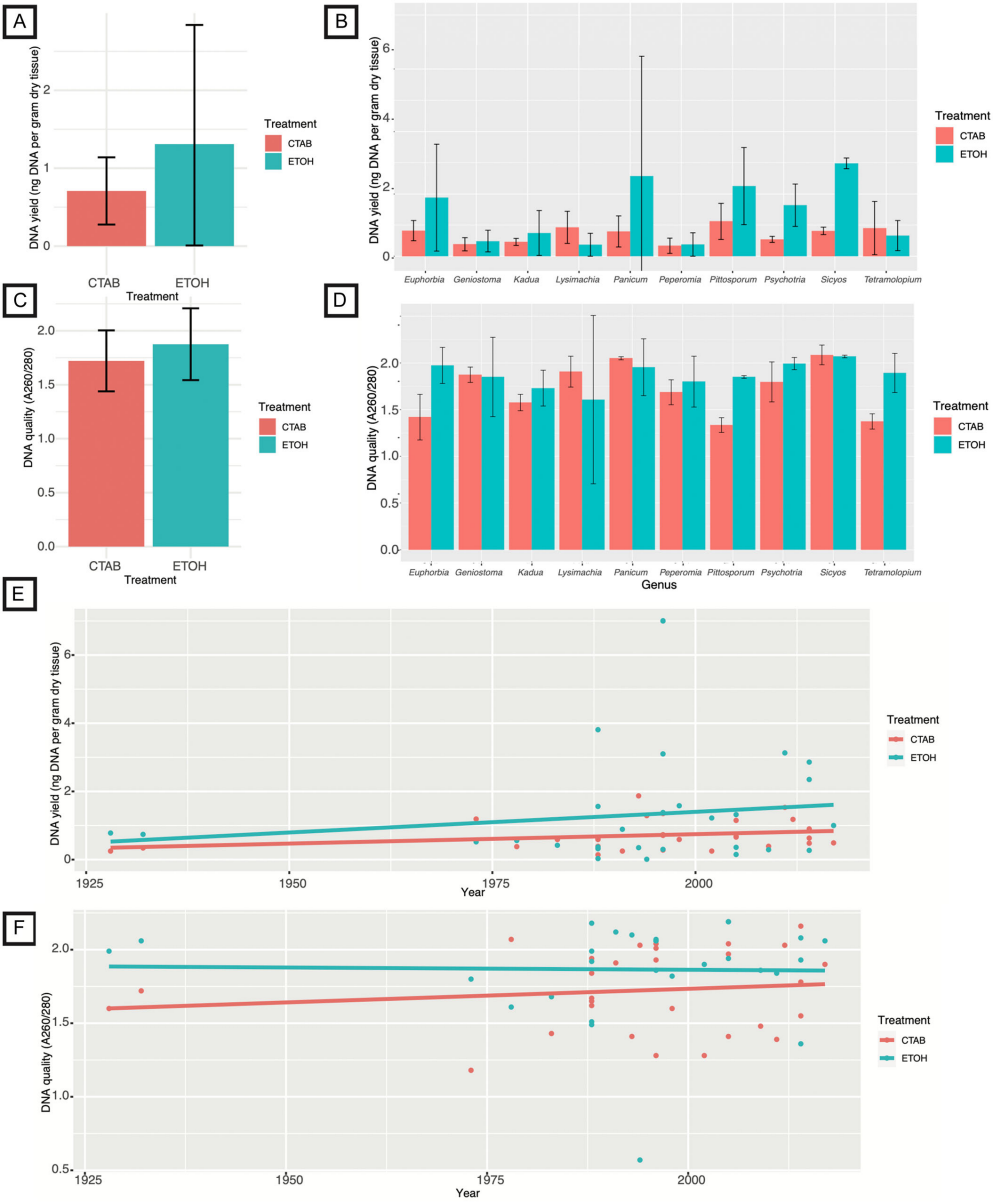
Quality and quantity of DNA from 10 endemic Hawaiian plant lineages derived from extractions performed using a CTAB protocol (CTAB) or a modified protocol with an ethanol pretreatment (ETOH). (A) Average DNA quantity as measured using a Qubit fluorometer. (B) Average DNA quantity as measured by Qubit fluorometer divided by genus. (C) NanoDrop‐derived average quality metrics for the DNA extracts. (D) NanoDrop‐derived average quality metrics for the DNA extracts divided by genus. (E) Associations between specimen age and DNA yield. (F) Associations between specimen age and DNA quality. Error bars indicate standard deviations.

**Figure 5:**
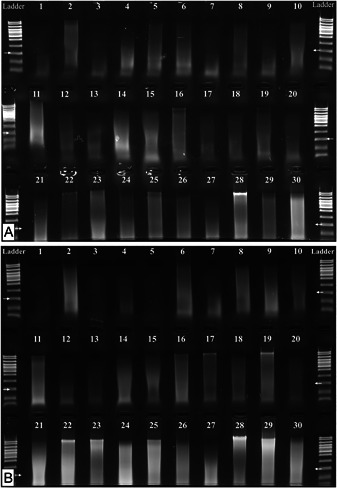
Gel electrophoresis separations of 30 DNA extracts derived from historical herbarium leaf tissues of endemic Hawaiian plant lineages. (A) Extractions prepared with the standard CTAB method. (B) Extractions prepared with the ethanol pretreatment method described in this study. Gel images of 2 µL of DNA stock were prepared using a 1.0% agarose LE gel. Arrows indicate the 300‐bp‐ladder band; any smaller DNA fragments (below this band) cannot be used to prepare a conventional Illumina library for Hyb‐Seq (i.e., target enrichment with genome skimming; Weitemier et al., 2014).

DNA yields from the historical herbarium tissues showed asymmetries between our two extraction approaches, which entailed the use of a traditional CTAB procedure or a modified protocol with an ethanol pretreatment. One sample of *Sicyos cucumerinus* A. Gray extracted using the ethanol treatment was conservatively removed from all analyses because the yield exceeded that of all other samples by an order of magnitude. The average DNA yield per milligram of dried herbarium tissue across all the Hawaiian plant lineages was slightly higher in the ethanol treatment than in the traditional CTAB approach, although meaningful variation across lineages was also evident in the resolution of a large standard deviation and a non‐significant difference between treatments (paired *t*‐test, *t* = −1.57, *df* = 28, *P* = 0.1277) (Figure 4A, Appendix S1). In two out of 10 lineages, namely *Lysimachia* L. and *Tetramolopium* Nees, the ethanol pretreatment was associated with a decrease in DNA yield. Once the variable effect of genus was accounted for, the extraction protocol was found to significantly affect DNA yield (two‐tailed ANOVA, *df* = 1, *F* = 4.42, *P* = 0.04), with an increase in yield associated with the ethanol treatment (Figure 4B, Appendix S1). On average, the ethanol pretreatments did not significantly increase the extract quality, as assessed using spectrophotometry (paired *t*‐test, *t* = −1.91, *df* = 28, *P* = 0.6) (Figure 4C, Appendix S1), although when genus was accounted for, this effect was statistically significant (two‐tailed ANOVA, *df* = 1, *F* = 4.16, *P* = 0.048) (Figure 4D, Appendix S1). Overall, we detected only a very minor association between sample age and DNA yield and quality, neither of which were statistically significant for either treatment (linear regression, *df* = 54, *r* ^2^ [concentration] = 0.02, *P* [concentration] = 0.26, *r* ^2^ [quality] < 0.001, *P* [quality] = 0.79) (Figures 4E, 4F; Appendix S1). The fragment length distributions of the ethanol‐treated samples are visible (Figure 5), showing that ample DNA at the ideal length distribution for NGS (typically greater than ~300 bp) was obtained for 20 out of 30 samples. Although none of the DNAs shown in Figure 5 were sequenced, numerous such libraries have been successfully generated and sequenced for other herbarium DNA extracts that produced similar patterns of fragmentation in gel electrophoresis (data not shown).

## CONCLUSIONS

In this study, the ethanol preservation or pretreatment of leaf tissue has been demonstrated to improve the quality of DNA isolated from species of the Vitaceae, red mangroves, and a variety of herbarium samples. Ethanol desiccation is certainly not the ideal preservation medium for all plant taxa at all physiological stages, and is by no means a replacement for silica gel; however, for the Vitaceae and red mangrove tissues in this study, ethanol treatment was the most optimal choice. While the ethanol pretreatment of the herbarium fragments may have assisted with the isolation of their DNA, these experiments cannot disprove that the action of the SDS‐proteinase K lysis buffer alone was the causal factor that improved the extraction compared with the CTAB method.

Collectively, these findings point to a need for plant researchers to consider testing different tissue preservation and/or pretreatment methods at the early stages of a research project to determine those best suited for the taxa under study. In addition to the ethanol treatments tested here, many extraction protocol modifications in the literature have been developed to address other taxon‐specific needs, such as thick cuticular waxes (Flournoy et al., 1996), deleterious polyphenols (Aleksić et al., 2012), copious polysaccharides (Tel‐zur et al., 1999; Russell et al., 2010; Shepherd and McLay, 2011), and PCR inhibitors (Štorchová et al., 2000), to name just a few. The DNA integrity within leaf samples should be given greater consideration during harvesting and storage before any extractions are conducted.

## AUTHOR CONTRIBUTIONS

G.J., S.W.J.C., I.H.L.M., W.W., and J.W. conceived the research and designed the experiments; G.J., S.W.J.C., and I.H.L.M. performed all the experiments. I.H.L.M. conducted the statistical analyses. S.W.J.C., I.H.L.M., W.W., and J.W. obtained the sample materials. S.W.J.C., I.H.L.M., W.W., and J.W. acquired the funding and analyzed the data. G.J., S.W.J.C., and I.H.L.M. wrote the manuscript. All authors approved the final version of the manuscript.

## Supporting information


**Appendix S1**. Data that were analyzed statistically to produce Figure 4. Columns A through M contain collection information related to the herbarium samples used in the experimental DNA extractions. Columns N through AB enumerate the quality and quantity of DNA extracted from each specimen using the standard CTAB protocol. Comparative data were collected for DNAs extracted using the newly developed method involving ethanol pretreatment, sorbitol wash, and proteinase K digestion; these data are listed in columns AF through AT.

## Data Availability

All data are available with the published article and Supporting Information.
